# *“When People Reach Out that is When They’re Desperate”*: Understanding Informal and Formal Help-Seeking Practices for Gambling among Aboriginal Peoples in the Northern Territory, Australia

**DOI:** 10.1007/s10899-024-10371-x

**Published:** 2025-01-25

**Authors:** Himanshu Gupta, Noemi Tari-Keresztes, David Aanundsen, James A. Smith

**Affiliations:** 1 Institute of Health and Management Pty Ltd., Charles Darwin University Campus, 2/187 Boundary Rd, North Melbourne VIC 3051, PO Box U362, Casuarina, NT 0815 Australia; 2 Flinders Health and Medical Research Institute, Rural and Remote Health, Flinders University, Charles Darwin University, PO Box U362 PO Box 42500, Casuarina, NT 0815 Australia; 3 The Fred Hollows Foundation, Indigenous Australia Program, PO Box U362 PO Box 42500, Casuarina, NT 0811 Australia

**Keywords:** Gambling, Australia, Help-seeking, Indigenous peoples, First nations peoples, Gambling policy

## Abstract

This study provides an in-depth qualitative exploration of Aboriginal peoples’ experiences with seeking help for gambling-related issues in the Northern Territory (NT), Australia. Through semi-structured interviews with 29 participants, including regular and occasional gamblers as well as those affected by others’ gambling, the research highlights key barriers to seeking formal help. These barriers included the normalisation of gambling within Aboriginal communities, denial of gambling problems, feelings of shame, privacy concerns, and a lack of trust in mainstream services. Additionally, past negative experiences with services, fear of judgment, and logistical challenges, such as long waiting times and transportation difficulties in remote areas, contributed to the low uptake of professional services. Instead, informal support from family and friends was occasionally sought, reflecting the collectivist nature of Aboriginal cultures. Participants also reported employing self-help strategies and offered practical suggestions for minimising gambling harm. This research underscores the complexity of gambling behaviours within Aboriginal communities and the cultural, social, and systemic factors that deter access to formal support services. It calls for the integration of Indigenous knowledge and practices into gambling prevention and intervention programs, which may improve the relevance and effectiveness of these strategies. By addressing both cultural norms and access barriers, such targeted approaches may reduce the need for reactive interventions and better support the health and wellbeing of Aboriginal people affected by gambling in the NT. To improve relevant policies and practices, we also consider these findings to contribute to the broader Indigenous-specific gambling prevention evidence-base contexts nationally and globally.

## Introduction

### Gambling Landscape in the Northern Territory, Australia

Gambling in the Northern Territory (NT) has seen both declining overall participation and increasing risks among problem gamblers, especially with regards to Electronic Gambling Machines (EGMs, colloquially called pokies or pokie machines). From 2015 to 2018, the number of people classified as moderate-risk or problem gamblers rose significantly, reflecting an increase from 1,200 to 2,500 individuals during this period (Stevens, 2020).

By 2018, about 4% of NT adults, or approximately 6,400 people, were identified as either moderate-risk or problem gamblers, despite a decline in overall gambling participation (Stevens, 2020). Furthermore, 8% of NT adults, or approximately 14,500 people, reported experiencing negative consequences from someone else’s gambling during this time (Stevens, 2020).

The gambling harm disproportionately affects the Aboriginal and Torres Strait Islander peoples (respectfully Aboriginal^1^ hereon) in the NT. Gambling is widespread among Aboriginal peoples, but factors such as a history of gambling, significant socioeconomic disadvantage, and sociopolitical challenges have led to different gambling motivations and behaviours compared to non-Aboriginal peoples (Consulting et al., 2017; Davidson et al., 2018).

Like others, Aboriginal peoples gamble on casino games, card games, horse races, and sports. However, regional variation in gambling activities among Aboriginal peoples occur in the NT. For example, card games are more prevalent in remote NT while pokies are more popular in urban NT where availability and accessibility to casinos and licensed venues is greater (Stevens, 2020).

### Problem Gambling and Gambling Risk

Significantly higher rates of problem gambling have been reported for Aboriginal peoples in the NT than other populations. For example, in 2015, about 4% of Aboriginal respondents were classified as moderate-risk or problem gamblers compared to 1-3% of non-Aboriginal respondents. By 2018, this prevalence had risen, with regional variation ranging from 6 to 13% across NT for Aboriginal respondents compared to 3-4% for non-Aboriginal people (Stevens et al., 2017).

Aboriginal Territorians also experience notably higher rates of problem gambling risk and harm from someone else’s gambling compared to non-Aboriginal populations. In 2015, harm from someone else’s gambling among Aboriginal respondents ranged from 11 to 78%, and this decreased to 5-26% by 2018. In contrast, the rates for non-Aboriginal respondents were much lower, ranging from 6 to 12% in 2015 and 5-6% in 2018 (Stevens, 2020; Stevens et al., 2017).

The above-mentioned regional variations in gambling behaviour and harm among Aboriginal peoples in the NT are influenced by a combination of factors, including access to different types of gambling products and distinct social contexts. For example, in some remote Aboriginal communities, card games are more popular due to their cultural relevance and accessibility, because of the collective nature of card games.

In contrast, in urban and regional areas pokies are more prevalent, leading to higher exposure to different forms of gambling (Stevens, 2020; Young et al., 2011; Young et al., 2008). When Aboriginal people from remote areas travel to urban centres, they may engage in different gambling behaviours, such as playing pokies, which are unavailable locally (Gupta, 2021). These shifts in gambling behaviour highlight the importance of understanding how access to gambling products and local social contexts contribute to the risk of problem gambling and harm in different regions.

The significant variation across regions underscores the disproportionate impact of gambling harm on Aboriginal communities in NT. It also shows the necessity for a deeper understanding of the regional differences in gambling behaviour and its associated harms among Aboriginal people in the NT. This understanding is crucial for developing targeted interventions that address the unique cultural and social contexts within these communities.

### Help-Seeking for Gambling Issues

In the NT, help-seeking for gambling issues among Aboriginal peoples is often not proportionate to the level of harm experienced, and prevention strategies involving health and social services are rarely sought. In addition, there are several barriers that prevent Aboriginal people from seeking professional help for gambling-related issues.

For instance, in many Aboriginal communities, gambling is socially accepted, and as a result, some individuals may not view their gambling as problematic. This acceptance reduces the perceived need to access gambling support services, making intervention efforts even more challenging. Even when severe harms are evident, help-seeking remains low, largely driven by cultural factors including feelings of shame and embarrassment, and a lack of awareness about available professional services.

In many instances, Aboriginal people are also hesitant to engage with mainstream gambling support services, which may not align with their cultural practices or experiences. Geographic isolation and the subsequent limited access to gambling help services is also a deterrent to pursuing help, especially for those living in remote NT (Fogarty, 2018; Gupta, 2021; Stevens & Bailie, 2012).

### Self-Help Strategies

Many gamblers implement self-help strategies to manage their gambling problems. These strategies typically involve creating barriers to spending or accessing money, such as carrying a limited amount of cash to gambling venues, avoiding carrying credit cards, self-exclusion from gambling venues, having a partner or family member take control of finances, and setting up direct debits to ensure bills are paid before the gambler can access the funds (Davidson et al., 2018; The Victorian Responsible Gambling Foundation, 2012).

Other strategies include engaging in activities to consciously distract from gambling such as joining local sports clubs for social interaction or finding alternative ways to overcome boredom and loneliness (The Victorian Responsible Gambling Foundation, 2012). However, there is mixed evidence about the effectiveness of such strategies (Gupta & Stevens, 2021). To our knowledge, no such evidence exists in relation to Aboriginal peoples in the NT.

### Current Study

This research paper is based on a broader study (Gupta, 2021) that was conducted to address the scarcity of qualitative gambling research among Aboriginal peoples in the NT. This paper focuses specifically on Aboriginal peoples’ perspectives regarding help-seeking for gambling issues and self-help strategies they use to manage their gambling problems in the NT.

The study primarily involved Aboriginal people living in urban and regional areas of the NT. However, the findings are expected to have broader implications for Aboriginal people across the NT, considering the diversity among Aboriginal peoples throughout the region (Gupta, 2021). We also consider these findings contribute to the broader Indigenous-specific gambling prevention evidence-base contexts both nationally and globally. The sample included both gamblers and individuals who were negatively affected by someone else’s gambling.

## Methods

### Sample

A purposive sampling method was used to recruit participants from the 2015 and 2018 NT Gambling Prevalence and Wellbeing Surveys. Additional recruitment was conducted using a snowball sampling strategy, where initial participants referred others. We contracted a market research company to assist in recruiting regular (weekly) and non-regular (monthly) pokies, sports, and racetrack gamblers, as well as individuals negatively affected by others’ gambling. The company initially contacted 57 eligible respondents and shared their contact details with the research team. The lead researcher (HG) followed up with these individuals to organise interviews. Further recruitment (10 participants) was done via word-of-mouth. Factors such as age, sex, and region were considered during participant recruitment.

Of 57 eligible respondents, 29 people agreed to participate in the study, consisting of 15 pokies players, four sports/racetrack bettors, and nine individuals affected by others’ gambling. Of these, 10 participants were male, 27 were 35 years or older, and 17 were from the Darwin/Palmerston region.

### Data Collection

Given the unique cultural and demographic context of the NT Aboriginal population, we designed the study using a strength-based approach. Based on a similar study conducted in another Australian jurisdiction (Breen et al., 2010; Fogarty, 2018), the study design and interview schedule were adapted to the NT context in consultation with local Aboriginal stakeholders. The stakeholders included Aboriginal gamblers, those affected by others’ gambling, an Aboriginal research team member, and members of an Institutional Aboriginal Advisory Committee. This consultation ensured the interview content was respectful and culturally appropriate.

Three interview guides were developed, one for each participant category (pokie players, other bettors, and affected by others’ gambling), consisting of both common and category-specific questions. All interviews were conducted by an Aboriginal project team member (DA), trained in qualitative research. Darwin/Palmerston-based participants had the option of face-to-face or telephone interviews, with most preferring phone interviews to maintain anonymity and comfort.

Telephone interviews allowed the research team to cover a wider geographic area, with 24 interviews conducted via phone and five conducted face-to-face. Face-to-face interviews (*n* = 5) were conducted at a university office. Qualitative data collected via telephone are regarded as equally robust and valid compared to face-to-face interviews (Sturges & Hanrahan, 2004; Sweet, 2002). This is especially true when dealing with sensitive topics, such as gambling (McCoyd & Kerson, 2006; Novick, 2008). Conducting interviews over the phone also offered participants greater anonymity, which made them more comfortable and willing to disclose intimate and sensitive information.

All interviews were audio-recorded with participants’ consent, and each interview lasted approximately 30–45 min. At the beginning of each telephone interview, the participant information sheet (PIS) was read aloud to the participants and verbal consent was obtained and recorded. For face-to-face interviews, participants were provided with a hard copy of the PIS and written consent was collected. A professional transcription service was used to transcribe all the audio recordings from the interviews.

Participants received a $50 grocery voucher for their time which excluded the purchase of alcohol, tobacco, or gambling products. The interviews explored participants’ experiences (in the past 12 months) of, and views on, help-seeking for gambling issues, including from personal contacts (e.g., partners, other family members, friends, work colleagues), formal services (e.g., General Practitioners (GPs), counsellors, welfare organisations, financial counsellors), and third parties (e.g., venue staff). Participants also expressed their views on self-help strategies they used, or others may use for managing their gambling issues. The datasets supporting the conclusions of this article are included within the article.

### Data Analysis

The interview data were analysed using an open coding technique, combining both deductive and inductive approaches to develop the coding framework (Gale et al., 2013; Parkinson et al., 2016; Ritchie & Lewis, 2003). We considered this approach appropriate because it allowed us to integrate both pre-existing theoretical constructs (a deductive approach) and emergent data-driven themes (an inductive approach) in developing the coding framework. This dual approach aligned with our study objectives, as it enabled us to explore specific areas identified in the literature (Breen et al., 2010; Gupta & Stevens, 2021) while also discovering unexpected themes and insights within the data. We commenced this process by identifying existing themes and then combining these with those that emerged from our data. For example, the reluctance to seek professional help was driven by shame, a desire for privacy, and a lack of trust in the effectiveness of mainstream services (pre-existing theme). This theme was combined with a lack of immediacy of help and transportation difficulties (a theme that arose from our study).

Two researchers (NTK and DA) coded the data. DA identifies as Aboriginal, which ensured cultural sensitivity during the data analysis process. Two local Aboriginal stakeholders were also consulted to refine the framework and ensure accurate representation of participant voices.

Both researchers resolved any differences that arose during the coding process through discussions and worked collaboratively to develop the final coding framework. This collaborative approach ensured that the data were accurately interpreted, and that the final coding framework was comprehensive and reflective of the themes identified in the interview data. We used QSR NVivo 12 software for data management. Verbatim quotes from participants were included in the results, with references made to their sex, age group, and region, as appropriate.

### Ethics

The study received ethics approval from the Central Australian Human Research Ethics Committee (CA-20-3747) and the Human Research Ethics Committee of the Northern Territory Department of Health and Menzies School of Health Research (2020–3728).

## Results

### Barriers to Seeking Help for Gambling Issues

Most Aboriginal gamblers involved in our study were aware of available support services; however, few actively sought professional help. Multiple reasons were reported for why awareness did not translate into action. The disconnect between recognising a problem and taking action may suggest deeper psychological, social, or financial challenges that prevented individuals from pursuing help.

The reluctance to seek professional help was driven by shame, a desire for privacy, and a lack of trust in the effectiveness of mainstream services. A common theme was the denial of having a gambling problem, with several participants feeling that their gambling was under control and did not warrant professional intervention. Others were reluctant to seek help due to embarrassment or fear of being judged. There also seemed to be a tension between different participants in whether they were or were not willing to speak to their friends or families about their gambling issues. It was particularly mentioned in the context of being a male and the associated societal perceptions of masculinity, as participants put it:

*“Pride might be a good one*,* again with that whole idea of that stigma within the [Aboriginal] community*,* within the family that you’re supposed to be the provider… If you’re the only person working within that household*,* that’s a lot of responsibility*,* and then that creates that cycle too….Once you start breaking it down*,* you can see why a lot of mob don’t like talking or reaching out within a family group about gambling or even drugs and alcohol too because there’s that stigma of you’re supposed to be the breadwinner*,* you’re bringing the money in*,* it’s your responsibility to look after us*,* and you just spent all that money.”* (M, 25–34 years, affected other, Darwin/Palmerston).

*“It comes down to that stigma as well*,* like “oh*,* if I’m gambling all my money*,* I must be a loser*,* or I must be a real dickhead. I’ve got a wife and kids or a husband and kids to look after or a carer for my grandmother or mother like shit*,* I’m just wasting all my time*,* money on bullshit*,* really in a sense”.* (M, 25–34 years, affected other, Darwin/Palmerston)

The normalisation of gambling within Aboriginal communities emerged during the conversations. Some participants described that gambling had become a routine activity within their social circles, making it difficult to recognise as a problem. One participant stated:

*“…if you’re gambling with your friends and your family*,* it’s very hard to pull up because it’s become normalised behaviour in a lot of areas*,* particularly around Pokies for urban people and more so cards and things*,* cards on tarps out in communities. And*,* so*,* it’s being done in big groups. So*,* family and friends aren’t necessarily going to pull people up…”* (F, 45–54 years, Alice Springs).

Participants described that in instances where there were other concurrent issues and addictive behaviours to deal with (alcohol, drugs, food security etc.), seeking help only for gambling could be limiting. Feeding other addictions and a clouded mind from simultaneous issues could also affect pursuing help for gambling issues, as gambling may not be seen as a priority issue to get addressed. Another participant added:

*“…if you’re comparing circumstances of losing if they’re [gamblers] having so many problems*,* whether they talk about it around helplessness or stigma associated with being stereotyped or labelled. Probably don’t feel much like a person*,* and they are in need of help; more like someone to study.”* (F, 45–54 years, affected other, Alice Springs).

Participants also reported feeling uncomfortable initiating conversations about gambling and discussing the problem openly, even with close family and friends. There was a strong desire for strategies to reduce stigma and create safe spaces for open dialogue:

*“So just bring gambling into the normal conversation with a person who*,* for example*,* you see that has got a gambling problem or an issue. Because people are not comfortable or they don’t know how to bring that stuff into the normal conversation*,* and hence they can’t really help.”* (F, 45–54 years, affected other, Alice Springs).

*“…making people feel comfortable about talking about gambling?… I don’t know. I think it’s a really hard thing to make anyone feel comfortable about something that may have hurt them or caused trauma or they may feel shame about.”* (F, 35–44 years, affected other, Darwin/Palmerston).

Instances were described where gambling affected gamblers and their families in some way, regardless of the intensity of the problem. While some expressed frustration at being unable to convince the gambler to seek help, others highlighted the emotional toll of supporting a gambler. One participant stated:

*“It hasn’t impacted on me in the case that my family go without food*,* or rent doesn’t get paid*,* or the mortgage doesn’t get paid. But it still creates friction and arguments because I have to be the one that says*,* “Stop*,* stop using our money.”* (F, 35–44 years, affected other, Alice Springs).

In some cases, affected others had to resort to indirect methods, such as providing financial assistance or separating themselves from the gambler. One participant shared their experience of slipping self-help cards into a family member’s purse, only to be met with anger.

Participants highlighted logistical and procedural challenges in accessing formal support services. These included long waiting times and the complexity of enrolling in services. In many cases, these challenges dissuaded participants from following through with their help-seeking attempts, as one participant described:

*“You’ve got to actually reach out*,* get triaged*,* do an intake*,* make an appointment. Sometimes when people decide to reach out on any sort of problem*,* they want that immediate help*,* and then if they’re given too long having to wait for it*,* then they move on from it or talk themselves out of it*,* or the moment passes it. So*,* I think for a lot of services in general*,* that time it takes to get an appointment and have any meaningful conversation is a real barrier.”* (F 35–44, affected other, Darwin/Palmerston).

Participants also highlighted transportation difficulties and lack of direct access to services, especially in remote communities. It was also reported that many Aboriginal Territorians from remote regions travel to urban centres and indulge in risky behaviours, such as gambling and other addictions. The problem arise when they go back to remote communities where there is a lack of gambling harm support services and thus unable to get help. One participant noted:

*“The thing is*,* with these services [health services]; yes*,* they’re there*,* but it’s a mission to actually get there when you need it…. if you don’t have a car*,* you’ve got to catch a bus*,* and if you got kids – you get turned off getting the help if you’re already struggling with daily life. I think if someone could come to your house there and then*,* that I reckon would do wonders*,* but the fact that a lot of people – in my situation*,* if I was told I was getting an appointment and then I had to be here at this time on this day*,* by then I’m over it. I don’t want to get your help. I’m trying to deal with a one year old and a few months old*,* and I got no car*,* and you want me to get to you? Good luck. It’s just easier for me to keep gambling. A lot of people want the help*,* but it’s just the services are sometimes just a hell of a lot just to even get into.”* (F, 35–44 years, gambler, Alice Springs).

Additionally, some participants described negative past experiences in seeking help from health and social services, which discouraged further attempts to seek assistance. For instance, one participant explained how her experience with a support service in a different jurisdiction dissuaded her from pursuing further help:

*“Yeah [I got help]. That was [organisation name and place excluded for anonymity]. Even though I wanted it [help]*,* like I said*,* until I was forced into a corner and actually was just confronted about it*,* I wouldn’t have actually*,* I think*,* gone until I was absolutely*,* yeah*,* like I said*,* homeless. It’s because I wanted it*,* I guess I didn’t know how to do it*,* and then you’re embarrassed. Like I said*,* I reached out once but then I was told*,* we’re going to get your number*,* and we’ll call back and all this shit; so*,* then I gave up. So that turned me off. I actually did reach out for help before I got that counselling*,* but I was turned off from the way in which it was handled*,* and so then until I was forced in the corner*,* I went to this counselling. I would have preferred to just be able to call and just talk to someone at that very moment I made that phone call.”* (Female, 35–44 years, gambler, Alice Springs).

The above example also talks to the immediacy of help that is required or desired but not addressed. Dovetailing with the fear of judgement by professional services, another participant emphasised the importance of timing of receiving appropriate help, particularly when individuals were in crisis:

*“Or they always need a referral from a doctor*,* and it’s like*,* people don’t want to be telling everyone their business. I’m calling you directly because I’m desperate and I need to talk to someone now or I’m going to do something stupid. That’s a lot of the time when I make the phone call. But most of the time you may be calling some business and it’s like*,* “Can I take your name*,* your number*,*” or “Have you got a referral?” It’s just*,* you hang up before they even finish because you need that direct help there and then. And when people reach out that is when they’re desperate*,* and they desperately want help. So*,* like I said*,* if it doesn’t come through there and then*,* they’re going to be turned off and probably won’t look for help again.”* (Female, 35–44 years, gambler, Alice Springs).

### Influence of Underlying Issues

Many participants emphasised that gambling was often a coping mechanism for deeper underlying issues, such as family problems, substance use, or mental health challenges. They stressed the importance of addressing these core problems before tackling the gambling itself. These conversations speak to a greater focus on social, cultural, economic and commercial determinants of health when developing gambling harm reduction support services for Aboriginal peoples. It is reflected in the below participant quotes:

*“I think it depends on the core situation of the individual*,* like why are they gambling and what’s the reasons behind that distraction to life? Whether it’s family problems*,* domestic violence or children*,* work. Are they doing it as a substitute for something else they could be trying to give up*,* smoking and drinking*,* so they turn to something else to distract themselves instead? Or they might do it because they drink a lot*,* so they also want to gamble because they’re drinking and smoking and doing drugs*,* so they have that need to gamble…if that person’s gambling as a distraction from family*,* well then there’s obviously a family issue that is husband and wife arguing and carrying on while the kids aren’t participating in house duties and are playing up at school or vice versa….if it’s family-related*,* they need to talk to counsellors*,* seek that core.”* (M, 25–34 years, affected other, Darwin/Palmerston).

*“I guess she [counsellor] helped me to understand that a lot of the reason people gamble is what she called an iceberg effect. Gambling is at the top*,* but there’s all this trauma and all these things that haven’t been diagnosed or touched on underneath the iceberg. That’s how she explained it to me. It’s also relating your emotions to actions*,* and a lot of it was just being consciously aware of what’s setting you off. What makes you react like that? Because there was always an emotional reaction to why I went and gambled.”* (F, 35–44 years, gambler, Alice Springs).

### Appropriate Support Networks

Participants had varying views on who was best suited to intervene or help gamblers with their gambling issues. Some expressed professional services were appropriate, with one participant mentioning that even though her GP was good, she never felt confident and comfortable talking about her gambling issues with her GP:

*“I did have a real deadly doctor. I liked him but*,* yeah*,* funny as I was comfortable talking to him about absolutely everything else but no*,* I wouldn’t talk to him about gambling.”* (F, 35–44 years, gambler, Alice Springs).

There appeared to be a consensus that GPs were not specialised in dealing with gambling addiction, which made them less effective in providing the necessary support. One participant simply remarked:

*“GPs can’t help… they don’t specialise in gambling and addictive behaviour.”* (M 25–34 years, gambler, Darwin/Palmerston).

*“To receive the support when it comes to drugs or alcohol or even domestic violence*,* it’s very apparent in the [Aboriginal] communities where to go and who to ask for assistance and help*,* to get maybe some support if you want to try and give it up or change your way. When it comes to gambling*,* it’s not apparent who do you go to. Who do you ask? You don’t want to go and ask your doctor*,* they’re for medical purposes. You don’t want to go and ask [a relationship support provider] that’s about your personal stuff. You don’t want to ask*,* you know*,* who do you ask?”* (F, 65 + yrs, affected other, Darwin/Palmerston).

For those who did access support, counselling services were seen as beneficial. One participant recounted:

*“My counsellor explained it to me that gambling is also relating your emotions to actions*,* and a lot of it was just being consciously aware of what’s setting you off. What makes you react like that? Because there was always an emotional reaction to why I went and gambled.”* (F, 35–44 years, gambler, Alice Springs).

Participants largely agreed that family and friends played a crucial role in recognising early signs of problem gambling. However, not all family members were equipped to handle the situation. They noted the comfort and safety found in talking to family and friends, but also acknowledged that professional help might be necessary in more severe cases, as one participant shared:

*“I have tried to support my friends through their stuff [gambling issues]. But I’m not equipped enough for it… God*,* you wouldn’t tell your work colleagues*,* would you?”* (F 35–44, gambler, Darwin/Palmerston).

Aboriginal cultures are often regarded as collectivistic. As such, a collectivistic approach was often described as an effective mechanism to address problem gambling. This highlights the importance of unified family interventions in Aboriginal contexts. One participant noted:

*“I know with my dad*,* it was his sisters that ended up approaching him*,* but they did it all together. I guess it helps to have the numbers and have that support because it’s easy to brush off what one person says*,* but a whole - all his sisters sort of had a unified approach.”* (F 35–44 years, affected other, Darwin/Palmerston).

Further, the comfort of family or friends was preferable due to the trust and familiarity involved. As one participant stated:

*“…as an Aboriginal person*,* that’s what we do. It’s part of life that you ask family for help and how family always helps. But that’s usually in the support and the scaffolding of when things are going pear-shaped.”* (F, 65 + years, affected other, Darwin/Palmerston).

To overcome the issues of judgement and stigma related to gambling, many participants described the role of Aboriginal Health Practitioners and Aboriginal community groups in providing professional help for gambling issues in a positive light:

*“It would be the Aboriginal Health Practitioners. … But it needs to be somewhere private and confidential. Those kinds of things.”* (F, 35–44 years, gambler, Darwin/Palmerston).

*“I suppose support groups within the community with Elders perhaps - probably community members that are significant in people’s communities that could encourage small groups I suppose.”* (F, 45–54 years, gambler, Darwin/Palmerston).

Services that involved people with lived or living experience of gambling issues were considered crucial in providing safe spaces for help-seeking:

*“I’ve found you’re more likely to talk to someone who has experience like you because you feel they know what you’re talking about*,* you know? Someone who even though they mean well*,* they may come up and try and talk to you and you think “Oh*,* what do you bloody know? Piss off. " So*,* but if you’re sitting down with someone who’s*,* look*,* just as downhearted as you*,* in the dumps as you*,* then you’re more likely to talk to them than someone who wants to help you.”* (F, 45–54 years, Darwin/Palmerston).

*“So*,* talking*,* like the Alcohol Anonymous*,* it’s talking it out*,* does help sometime… So*,* I think they should keep that kind of service open because when you hear other people go through problems*,* you also give a person possible solutions to their problems*,* their habits. So that really*,* really helps just talking out loud*,* like in a casual sort of environment*,* you know.”* (F, 45–54 years, Darwin/Palmerston).

### Self-Help and Coping Strategies

Discussions around self-regulation strategies revealed that many gamblers employed self-help strategies to manage their gambling. These included leaving credit cards with a partner, setting financial limits, and taking breaks from gambling peers. However, such strategies were not always effective, and in some cases, led to further tension or frustration. One participant shared:

*“…my ex did that to me loads of times. He said*,* “You take my card. Don’t give it back to me. Don’t you give it back to me.” But then*,* two hours later and six drinks later*,* it would be like*,* “Give me my card now…. But then*,* you can also end up bearing the brunt of their anger about it too*,* once they have changed their mind*,* and they are in a different headspace.”* (F, 35–44 years, affected other, Darwin/Palmerston).

Other participants found success in adopting methods used to overcome other addictions, such as spacing out gambling sessions or engaging in alternative activities like exercising, as one participant described:

*“…this trick I learned with a spacing thing. And actually*,* they used to have this good thing on TV*,* it was an ad about drinking alcohol and spacing it. And then*,* I started picking that – about*,* have one beer*,* then your next one*,* have a water. And so*,* you’re rehydrating. So*,* every second one was a beer*,* and every second one was water*,* so you’re rehydrating. And then*,* I started to do that a little bit with my gambling – maybe I’ll gamble – do a lotto ticket this week*,* then I’ll give next week a miss.”* (M, 45–54 years, gambler, Darwin/Palmerston).

*Or sometimes*,* what I’ll do is just keep myself occupied with things as well*,* too…So some of it*,* I’d start going to the gym and having other activities and stuff like that. So*,* looking for new activities and have something that would occupy my mind as well*,* too. So*,* I think that was it*,* and not being around – trying to have breaks from people who had the problem as well*,* too. So*,* I’m trying to hang out with different people*,* so I wasn’t always being influenced by gambling”* (M, 45–54 years, gambler, Darwin/Palmerston).

Some participants also spoke about excluding themselves from gambling venues to limit their gambling. For example, they reported how exclusion worked in other Australian jurisdictions and could be applied in the NT. These conversations reflected transience of gambling and the need for prevention strategies that permeate state/territory boundaries.

*“I don’t know if they do it up here*,* but down in Perth*,* the casino*,* you can ask to be blacklisted. If you had a gambling problem. I don’t know if they do it in venues up here or not.”* (M, 45–54 years, gambler, Alice Springs).

### Intervention Timing and Tactics

Participants generally agreed that intervention was necessary when gambling began to impact family members, finances, or health. As one participant put it:

*“I think when it starts - when the impacts have that ripple effect. So*,* by him losing money and him not being able to afford rent*,* then someone in the family having to chip in or take him in even has been the case. So*,* when it starts affecting others*,* when it starts affecting health or the ability to afford basic needs*,* and also when it - yeah*,* I guess this comes under affecting others. Like*,* missing family things because you’re stuck at the Pokies and losing track of time*,* or not going home and sleeping because you’re sitting at the casino and that sort of thing.”* (M, 25–34 years, affected other, Darwin/Palmerston).

Another participant emphasised that intervention should occur when a gamblers’ financial situation deteriorates:

*“I think when you know you’ve got no money left*,* and you have to ask somebody else for money*,* then there is a problem.”* (F 45–54 years, gambler, Darwin/Palmerston).

Strategies for approaching gamblers were also discussed. Some participants suggested that venue staff could play a role in intervening with regular gamblers. Gentle but assertive communication with gamblers in a silent and safe space by the venue staff was described as a potential approach with regular and/or problem gamblers for their gambling issues. Acknowledging gamblers’ challenges and underlying issues with problem gambling; however, should be the first step in the process. One participant noted:

*“Firstly*,* you have to know it’s a problem*,* don’t you? … The sticker – you don’t talk to it. … Then the venue people know who’s there every day. They might be able to just have that conversation. “You’ve been here five times this week…. I think it’s just a general conversation around*,* “I’ve observed you. Do you want to talk about anything?” You don’t need to get to the personal side of it. But observe what people are doing and then you can point out to people what they’ve been observing.”* (F, 35–44 years, gambler, Darwin/Palmerston).

Many participants described gamblers should be encouraged to take some sort of breaks (such as a tea break or a food break, hang out with people, etc.) between their gambling sessions. This might distract them from gambling and break the positive sensory reinforcement provided by the lights and sounds emitted by the pokie machines for endless gambling:

*“Maybe they could put people in the clubs that go around and just say hi to people when they’re playing*,* and just to see and maybe try and distract them for a little bit. If they’ve been sitting there for a long time*,* maybe talk to them*,* see if they want to go hang out or do something else. I don’t know. If you’re playing*,* you don’t want to stop playing.”* (F, 45–54 years, affected other, Darwin/Palmerston).

*“Seeing people flick on the screen…The smells*,* the lights*,* the sounds*,* it becomes a sensory overload too like “oh it’s so cool in here like my machine’s going off”.* (M, 25–34 years, affected other, Darwin/Palmerston)

Communication and other strategies likely play a significant role in approaching and supporting gamblers. The strategies reported by participants highlighted how gambling impacts the affected person instead of telling the gamblers what to do and changing and developing ways to deal with them:

*“I just found - when I talk to him [father] now about it and reflect on things*,* and still it comes up as a problem now and then*,* I use statements about how it impacts on me …So*,* I’ve stopped trying to do the whole*,* like*,* telling him what to do*,* but I say*,* “well when you do this*,* it impacts on me this way*,* or makes me want to pull away and not spend time with you” or whatever. So*,* I just make it about the impact on me rather than framing it as me telling him what to do.”* (F, 35–44 years, affected other, Darwin/Palmerston).

*“I’ve just learned to change my strategy in dealing with it. I made it quite clear I do not lend money anymore. I do not give money over because I know where the money goes. But she [daughter] has then changed her strategy. She’ll use her cash to gamble*,* and then next minute*,* the electricity’s disconnected*,* and she knows that I won’t let the electricity sit off because of the grandkids. So*,* then I’d say the next minute*,* well then*,* I change the strategy*,* okay*,* well I took all the kids home with me for a whole fortnight until she got her next pay when she can get her electricity on. But she [daughter] had to stay at her house with no electricity on*,* and I had the kids…so you’re constantly having to change your strategies and your movement or your way of thinking or dealing and supporting because they are very smart at manipulating and getting what they want.”* (F, 65 + years, affected other, Darwin/Palmerston).

*“I find personally when I talk with friends and that*,* talking it out with people. Not only telling people about your problem but you’re also I see it as thinking aloud. And sometimes when you talk aloud*,* you come up with possible solutions yourself. It’s just like “Oh wow*,* yeah*,* I’ve got to try that”*,* it’s that sort of thing for me. Yeah.”* (F, 45–54 years, Darwin/Palmerston).

Other strategies described by participants were asking how gamblers feel about their gambling such as acknowledging the feeling of disappointment and showing them the positive health and wellbeing impacts of not gambling:

*“Acknowledging how they are affected. Acknowledging how they might be feeling disappointed because there’s no money left*,* or the financial accounts aren’t looking too good for the next six months or whatever. And acknowledging all of that first and being able to work their concerns. That would help them begin to try and approach the issue objectively.”* (F, 45–54 years, affected other, Alice Springs).

*“The peer groups [might be best placed to approach gamblers]. Just organising things that don’t involve being at gambling would be really helpful. Family events that haven’t got gambling. Just fun and enjoying life and socialising that doesn’t include that*,* because people still need to feel connected and part of a community.”* (F, 35–44 years, affected other, Darwin/Palmerston).

In some cases, gambling was seen as a form of escapism from daily life challenges but having a conversation might help, as described by one participant:

*“It’s about - yeah*,* it’s mental framing of what it is. I know that*,* for Mum*,* we’ve a couple of conversations about it*,* and it is a relaxation space for her. So*,* the gambling isn’t the thing… I don’t think it’s ever been framed inside Mum’s head as an issue.”* (F, 35–44 years, affected other, Darwin/Palmerston).

## Discussion

This study offers a unique and in-depth perspective on the help-seeking behaviours and self-help strategies among Aboriginal peoples in the NT regarding gambling-related harm. The findings underscore the complexity of gambling behaviours within this population, highlighting how the collectivist nature of Aboriginal families, social stigma, and access barriers contribute to a low uptake of formal support services, among Aboriginal peoples in the NT.

While many participants in this study were aware of gambling support services, a significant gap existed between awareness and action. Several factors, including feelings of shame, denial of gambling problems, mistrust of mainstream services, and a lack of immediate help were identified as major barriers to seeking help (Breen et al., 2010; Fogarty, 2018). This is consistent with previous research that found stigma and embarrassment to be common deterrents to help-seeking among Aboriginal communities in other Australian jurisdictions (Hing et al., 2014).

Gambling is often normalised within Aboriginal social networks (Gupta et al., 2024), where it becomes embedded in daily routines, making it difficult for individuals to recognise it as a problem (Davidson et al., 2018; Stevens & Bailie, 2012). In our study, we identified that this normalisation of gambling in Aboriginal communities not only delayed help-seeking but also exacerbated the risks of gambling-related harm, particularly as many individuals resorted to gambling as a coping mechanism for deeper issues, such as family stress, substance use, or mental health challenges​.

The cultural context of Aboriginal communities plays a central role in how gambling is perceived and addressed (Breen et al., 2010). As identified in this study, traditional Aboriginal cultural values, such as collectivism and the importance of family, influenced how individuals perceived gambling and the role of social support. However, these same cultural factors created additional barriers to pursuing help when individuals feared judgment or damage to their reputation within their community. Geographic isolation in the NT further compounded the problem, limiting access to formal gambling support services, which were often not designed to be culturally responsive.

Self-help strategies among participants were varied, including financial management tactics (e.g., leaving credit cards with partners) and efforts to distract from gambling through alternative activities like sports or exercise. While these strategies provided short-term relief, they were not always effective in addressing the underlying issues driving gambling behaviour. Additionally, some participants reported using exclusion programs to limit access to gambling venues, but this strategy often required external support and enforcement​. Previous research conducted elsewhere has similarly found that while self-help strategies may mitigate some harm, they are insufficient without broader structural interventions, particularly for populations facing socio-economic disadvantage and geographic isolation (Breen et al., 2010; Davidson et al., 2018).

The study underscores the need for culturally tailored interventions that are accessible, engaging, and respectful of Aboriginal social and cultural practices; and address the unique challenges faced by Aboriginal peoples in the NT. Apart from the available mainstream services, Aboriginal communities in the NT might benefit by involving Aboriginal community-controlled services in gambling harm reduction support, as reported by our participants. Without such interventions, mainstream services will, therefore, likely continue to be underutilised by Aboriginal peoples in the NT (Stevens, 2020; Stevens et al., 2017). This calls for the development of culturally informed, community-based gambling harm reduction approaches that engage Aboriginal communities in designing and implementing interventions that resonate with their social and cultural contexts, ensuring they are relevant and trustworthy.

## Limitations

The following limitations should be considered when interpreting the findings and applying them to broader gambling intervention strategies for Aboriginal communities in the NT.

- Sample size and representation: The study included a relatively small sample of 29 participants from various regions in the NT. While purposive and snowball sampling methods were employed, the sample might not fully represent the diverse experiences of Aboriginal peoples in the NT, particularly those in more remote or isolated communities.
- Geographic and cultural variation: The study focused primarily on urban and regional areas. Although the findings were intended to apply more broadly across the NT, the unique experiences of Aboriginal peoples in remote communities were less explored. Regional variations in gambling behaviour and access to services might require more targeted research to address the needs of different communities.
- Participants’ age and gender: We had more representation from older (35 + years) and female participants in our study. Therefore, including younger and male participants in future studies could provide diverse insights into gambling behaviours and barriers across different age and gender groups.
- Self-reported data: The qualitative nature of the study relies on self-reported data, which is subject to recall bias and social desirability bias. Participants may have downplayed or exaggerated their experiences with gambling or help-seeking due to stigma, shame, or personal interpretation.
- Focus on individual experiences: While the study explored personal and familial experiences, it did not capture broader systemic or structural factors such as government policies, funding for gambling intervention programs, or the role of gambling operators. These external factors might also significantly impact gambling behaviours and help-seeking patterns in Aboriginal communities.

## Future Research

The study primarily focuses on participants’ recent experiences and perspectives. As a result, it does not address the long-term outcomes of help-seeking strategies, self-help measures, or intervention efforts. A longitudinal study would provide deeper insights into the efficacy and sustainability of these approaches over time.

Although the study was aimed at understanding Aboriginal gambling experiences, the exclusion of non-Aboriginal perspectives, such as service providers or other stakeholders, limits a comprehensive understanding of how gambling-related interventions are perceived and delivered from different viewpoints.

## Conclusion

Addressing gambling-related harm among Aboriginal peoples in the NT requires culturally tailored, community-driven interventions that respect and incorporate Aboriginal values. By fostering community ownership of solutions, reducing stigma, and improving access to services, these efforts could help mitigate the significant gambling-related harm experienced by Aboriginal communities in the NT. To improve policies and practices in addressing gambling-related harm among Aboriginal peoples in the NT, we offer below recommendations that have emerged from our study:

- *Culturally informed gambling support services*: There is a need to develop culturally appropriate gambling support services that reflect the unique values and experiences of Aboriginal peoples in the NT. Aboriginal Health Practitioners and Elders may play a central role in these services to ensure that they are trusted and respected by the community. Integrating traditional knowledge and practices into gambling interventions may enhance their effectiveness and increase engagement.
- *Community education and awareness*: Community-led initiatives are needed to raise awareness of gambling harms and reduce the stigma associated with seeking help. Culturally appropriate education campaigns that normalise discussions about gambling within Aboriginal families and communities may help reduce shame and facilitate early intervention. These campaigns should also highlight the collective responsibility of families and communities supporting individuals with gambling issues, reflecting Aboriginal values of community solidarity.
- *Peer support networks*: The establishment of peer support networks, especially those involving individuals with lived experience of gambling harm, could provide a more accessible and trusted avenue for help-seeking. Peer support groups could offer a safe space for individuals to share their experiences, learn from one another, and develop coping strategies in a supportive and non-judgmental environment​.
- *Mobile and outreach services*: Given the geographic isolation of many Aboriginal communities in the NT, expanding mobile and outreach services is essential for reaching remote Aboriginal communities in the NT. These services can provide immediate, on-the-spot counselling and support, reducing logistical barriers such as transportation and long waiting times. Mobile units could also work with local community leaders to ensure the services are culturally appropriate.
- *Holistic approach to address underlying issues*: Gambling interventions for Aboriginal peoples require a holistic approach by addressing the underlying factors contributing to gambling, such as mental health issues, substance use, and family dynamics. This approach acknowledges that gambling is often a symptom of broader social and emotional challenges and providing integrated services may lead to more effective support.
- *Strengthening family and community-based interventions*: Family interventions, aligned with Aboriginal cultural values of collectivism, should be promoted. Aboriginal communities might benefit from formal programs that train family members in how to approach and support individuals struggling with gambling issues​. Training family members to identify early signs of problem gambling and provide support could reduce denial and promote accountability. Aboriginal Elders and community leaders can play a crucial role in facilitating these conversations, ensuring that they are conducted in a culturally respectful manner.

## Data Availability

The datasets supporting the conclusions of this article are included within the article.

## Footnotes

1 The term ‘Aboriginal’ has used been used throughout the manuscript to reflect Aboriginal and Torres Strait Islander, Indigenous, or First Nations people, for purposes of brevity. We respect the diversity among these populations. Also, all participants in this study identified as Aboriginal and thus we have not included any reference to the Torres Strait Islander peoples in this paper.

## Data Availability

We have prepared this article based on our research study, “A qualitative investigation of Aboriginal gambling behaviour, consequences, risk factors, and help-seeking in the Northern Territory”. However, the analysis presented in this article is sufficiently different and provides new and unique insights into Aboriginal peoples’ views on help-seeking for gambling issues in the Northern Territory of Australia. Hence, this article provides new knowledge to the gambling literature. We have also cited the previous report in the article, where appropriate, to avoid any perception of self-plagiarism.

## Code Availability

QSR NVivo 12 software was used to manage the interview data.
